# A protocol to correct for intra- and interspecific variation in tail hair growth to align isotope signatures of segmentally cut tail hair to a common time line

**DOI:** 10.1002/rcm.7196

**Published:** 2015-04-27

**Authors:** Martina Burnik Šturm, Budhan Pukazhenthi, Dolores Reed, Oyunsaikhan Ganbaatar, Stane Sušnik, Agnes Haymerle, Christian C Voigt, Petra Kaczensky

**Affiliations:** 1 Research Institute of Wildlife Ecology, University of Veterinary MedicineVienna, Austria; 2 Smithsonian Conservation Biology InstituteFront Royal, Virginia, USA; 3 Great Gobi B Strictly Protected Area Administration & Department of Zoology, School of Biology and Biotechnology, National University of MongoliaUlan Bator, Mongolia; 4 Oslarija, Institute for Donkey Breeding and ResearchKomen, Slovenia; 5 Leibniz Institute for Zoo and Wildlife Research (IZW)Berlin, Germany

## Abstract

**Rationale:**

In recent years, segmental stable isotope analysis of hair has been a focus of research in animal dietary ecology and migration. To correctly assign tail hair segments to seasons or even Julian dates, information on tail hair growth rates is a key parameter, but is lacking for most species.

**Methods:**

We (a) reviewed the literature on tail hair growth rates in mammals; b) made own measurements of three captive equid species; (c) measured *δ*^2^H, *δ*^13^C and *δ*^15^N values in sequentially cut tail hairs of three sympatric, free-ranging equids from the Mongolian Gobi, using isotope ratio mass spectrometry (IRMS); and (d) collected environmental background data on seasonal variation by measuring *δ*^2^H values in precipitation by IRMS and by compiling pasture productivity measured by remote sensing via the normalized difference vegetation index (NDVI).

**Results:**

Tail hair growth rates showed significant inter- and intra-specific variation making temporal alignment problematic. In the Mongolian Gobi, high seasonal variation of *δ*^2^H values in precipitation results in winter lows and summer highs of *δ*^2^H values of available water sources. In water-dependent equids, this seasonality is reflected in the isotope signatures of sequentially cut tails hairs.

**Conclusions:**

In regions which are subject to strong seasonal patterns we suggest identifying key isotopes which show strong seasonal variation in the environment and can be expected to be reflected in the animal tissue. The known interval between the maxima and minima of these isotope values can then be used to correctly temporally align the segmental stable isotope signature for each individual animal. © 2015 The Authors. *Rapid Communications in Mass Spectrometry* published by John Wiley & Sons Ltd.

In recent years, segmental stable isotope analysis of hair has been a research focus of animal dietary ecology and migration.^1^–^7^ The isotope abundance of ^2^H and ^18^O in animal tissue (including hair) provides information on water intake, which is a reflection of the geographical location, and the isotope abundance of ^13^C and ^15^N provides information on diet and nutritional status.^8^,^9^ Tail hair is an ideal matrix for stable isotope analysis because it grows continuously in many species and is isotopically inert after formation.^10^–^12^ Consequently, when sampled and analyzed longitudinally, tail hair provides temporally explicit information on isotope abundance allowing inferences to be drawn on seasonal dietary regimes or movement patterns.^1^–^3^,^9^,^12^,^13^

To correctly assign hair increments to seasons or even Julian dates, information on hair growth rate is a key parameter. For most species such data is missing, scarce or suggests high intra-specific variation (e.g. horses; Table 1) and hence growth rates of related species are used as substitutes.^3^ However, this introduces considerable uncertainty as average growth rates, even between closely related species, may be different (Table 1) or if identical may be modulated by photoperiod, ambient temperatures, various hormones, nutritional status, and general health.^14^ Therefore, hair growth rates can be expected to differ among species, populations and even individuals of the same species living under different environmental conditions.

**Table 1:** Tail hair growth rates of different animal species obtained from the literature

Species	Growth rate (mm/day)	N	Reference
Domestic horse (*Equus caballus*)	0.63 – 0.88 (0.72 ± 0.03)*	6	31
	1.28	1	13
	0.83	29	32
Pony	0.79	5	30
Elephant (*Loxodonta africana*)	0.73 – 1.04	4	2
Cow (*Bos taurus*)	0.69 – 1.06	5	10
	0.51 – 0.63 (0.58 ± 0.01)	4	36

* mean ± SD

To convert monthly growth rates into daily growth rates, we used 30 days as the average number of days in a month.

In a study comparing the ecology of three sympatric equid species, domestic horses (*Equus caballus*), Przewalski's horses (*Equus ferus przewalskii*) and Asiatic wild asses (*Equus hemionus*) in the Mongolian Gobi, using segmental stable isotope analysis of tail hair we were confronted with a total lack of data on tail growth for the two wild equid species and an apparently high variability in average tail hair growth in the domestic horse (Table 1). Our measurements of tail hair growth rate in captive Przewalski's horses, domestic horses, and domestic donkeys (*Equus asinus*) confirmed inter- and intra-specific variation in tail hair growth and required us to explore alternative means of establishing the correct timeline for our tail hair increments.

In a highly seasonal environment the isotopic signatures of animal tissue can be expected to change in relation to changing environmental conditions, e.g. the freezing of surface water, availability of snow as a water source, or different availability and accessibility of certain food items.^15^,^16^ The aim of this paper is to present a simple and straightforward method on how to indirectly determine and correct for differences in tail hair growth rates based on the isotope signatures of segmentally cut hair in combination with the known interval length of environmental changes in a highly seasonal environment. The method can equally be applied to other species living in highly seasonal environments. The data presented here is primarily meant to illustrate the correction method; it is beyond the scope of the paper to present and discuss the full dataset and its implication regarding the comparative feeding ecology of the sympatric equids in the Mongolian Gobi.

## Experimental

### Study area in Mongolia

Samples from free-ranging equids came from the Dzungarian Gobi in and around the 9000 km^2^ Great Gobi B Strictly Protected Area (SPA) in SW Mongolia. The area consists of steppes, desert steppes and semi-deserts dominated by Chenopodiaceae such as the saxaul (*Haloxylon ammodendron*), a large shrub or small tree following the C_4_ photosynthetic pathway,^17^,^18^ Asteraceae such as *Artemisia* spp., and Poaceae such as *Stipa* spp., both of the latter following the C_3_ photosynthetic pathway.^19^ All three of these plant species are known to be important food plants for equids in the Dzungarian Gobi.^20^,^21^

The climate of the Great Gobi B SPA is continental with long, cold winters and short, hot summers. The average annual rainfall is 100 mm with a peak during summer. The average snow cover lasts around 100 days, but this varies and shifts throughout the winter. The area is generally considered to follow a non-equilibrium dynamics where biomass production and ungulate population fluctuations are both driven by the amount and the timing of rainfall events.^22^

The landscape is dominated by plains in the east and rolling hills in the west. The Altai Mountains flank the park to the north, and the Takhin Shar Naruu Mountains form the southern border with China. Elevations range from 1000 to 2840 m. Open water is unevenly distributed with almost no water in the central or western part of the park. In winter wildlife and livestock primarily cover their water requirements by eating snow.

### Tail hair measurements

To determine baseline values of tail hair growth rates in different equid species the tail hair growth rate was measured in five captive Przewalski's horses (♂ = 3, ♀ = 2, both mares not pregnant or lactating, age 4–8 years) trained to be handled in a custom-built chute system with a incorporated hydraulic restraint device (TAMER®; Fauna Research Inc., Red Hook, NY, USA) at the Smithsonian Conservation Biology Institute (Front Royal, VA, USA),^23^ three privately owned domestic donkeys (♂ = 1, ♀ = 2, 1 pregnant, 1 not pregnant and not lactating, age 3–6 years) in Slovenia, and a privately owned Lipizzaner horse (♂ = 1, age 14 years) in Austria. Unfortunately, no captive facilities with trained or tame Asiatic wild asses were available within the European Endangered species Program (EEP) network of zoos breeding Asiatic wild asses. All animal procedures at the Smithsonian Conservation Biology Institute were approved by the Smithsonian Institutional Animal Care and Use Committee (#13–34).

In each individual equid a 2×2 cm patch of tail hair was cut to skin level where the long tail hair originates. Subsequently, the length of the regrown hair was recorded roughly every 2–4 weeks and the growth rate determined in mm/day since cutting. Previous studies have suggested that there is no change in growth rate associated with shaving/cutting.^24^ The patches of regrown hair were recut every 6 weeks to facilitate discrimination of regrown from surrounding hair. All tail hair growth measurements were carried out between August 2013 and April 2014 and performed by the same person in the same way at each location.

### Stable isotope analysis

To assess the effect of known changes in the feeding and water regime we analyzed the tail hairs of two Przewalski's horses of similar age and same reproductive status (♀, not pregnant, not lactating, age 4 and 5 years) subject to identical housing and feeding regimes. Both Przewalski's horses were first held at the same captive facilities in Europe and then transported to the Mongolian Gobi on 19 July 2012 where they were kept together in a 160 ha adaptation enclosure until release on 11 June 2013. Both animals were sampled within 7 days around release time while anesthetized in order to attach a GPS radiocollar to facilitate post-release monitoring.^25^ As both animals simultaneously experienced the same dramatic environmental change, we expected their isotope signatures to change accordingly and their *δ*^2^H, *δ*^13^C and *δ*^15^N patterns to be highly synchronized.

For the stable isotope analysis of free-ranging equids in the Mongolian Gobi, several long tail hairs were plucked from temporarily restrained adult domestic horses and anaesthetized or recently deceased Przewalski's horses and Asiatic wild asses. Only adult animals (3–15 years) were selected for the study to avoid the effect of nursing on the stable isotope ratios in foals and yearlings. Since we are dealing with wild free-ranging animals, it was not possible to know whether the individual mares had been pregnant or lactating during the time period presented in their tail isotope profile (which basically goes back 2–3 years prior to sampling). Both sexes were equally presented for all species, with the exception of Asiatic wild asses (3:4). All tail samples were collected over a period of 4 years from 2009 to 2013 and stored in paper envelopes until analysis. Samples were imported from Mongolia into Austria in accordance with the Convention on International Trade in Endangered Species of Wild Fauna and Flora (CITES Nos. AT 13-E-1839 and AT 13-E-2193).

The timing of changes in the environmental conditions of free-ranging equids was inferred from the 16-day averaged normalized difference vegetation index (NDVI), a remote sensing product and proxy for pasture productivity^26^ acquired by the moderate-resolution imaging spectrometer (MODIS) at 250 m resolution and freely available from NASA's Earth Observing System Data and Information System (EOSDIS) via Reverb.^27^

Between June 2012 and August 2013, 26 precipitation samples for stable isotope analysis were collected at the Takhin Tal research camp located inside the study area (45°32'N, 93°39'E; 1760 meters above sea level (m asl)). The samples were collected from a rain collector as soon as possible after a precipitation event and transferred into 50-mL bottles with a tightly fitting cap and kept in a dark place until transport and analysis.

In the laboratory, any dirt or fat was removed from individual tail hairs by wiping them quickly with 95% ethanol prior to CN analysis and cleaning them with a 2:1 chloroform/methanol solution prior to H analysis. The hairs were subsequently dried in an oven at 50 °C and cut into 10-mm segments with a scalpel. We always used the longest hair of an individual for the combined CN analysis and the second longest hair for the H analysis. From each hair segment, a subsample of 0.35 ± 0.05 mg was enclosed into a tin cup (IVA Analysetechnik e.K., Meerbusch, Germany) for CN analysis and a subsample of 0.25 ± 0.05 mg into a silver cup (IVA Analysetechnik e.K.) for H analysis. Prior to H analysis, trays with prepared samples were stored in the lab for at least 1 week to equilibrate with air moisture.

Stable isotope analyses were conducted at the stable isotope facility at the Leibniz Institute for Zoo and Wildlife Research (IZW), Berlin, Germany. Carbon and nitrogen isotope ratios were determined simultaneously after combustion using a model CE1110 elemental analyzer (EA; ThermoFinnigan, Bremen, Germany) coupled in continuous-flow mode to a Delta Plus isotope ratio mass spectrometer (ThermoFinnigan). For the isotope analysis of the non-exchangeable hydrogen, the tail hair samples were placed into the autosampler (Zero Blank autosampler; Costech Analytical technologies Inc., Milan, Italy) of the EA (HT Elementaranalysator HEKAtech GmbH, Wegberg, Germany) where they were flushed for at least 1 h with chemically pure helium (Linde, Leuna, Germany) before pyrolysis at 1450 °C. The hydrogen isotopes were then measured on a Delta V Advantage isotope ratio mass spectrometer (Thermo Fisher Scientific, Bremen, Germany), connected via a Conflo III interface (Thermo Fisher Scientific) to the EA. The precision of the measurements was always better than 0.1 ‰ for the *δ*^13^C and *δ*^15^N values and 1.0 ‰ for the *δ*^2^H values, based on the repeated analysis of the laboratory standards (leucine and tyrosine for CN and three in-house keratin standards for non-exchangeable H in the hair). In-house laboratory standards were calibrated with the international standards NBS 19, NBS 22, USGS 24 and L-SVEC for carbon, IAEA-N1, IAEA-N2 and IAEA-NO3 for nitrogen and VSMOW and SLAP for hydrogen. Details of the in-house keratin standards are described in Popa-Lissenau *et al*.^28^

The *δ*^2^H and *δ*^18^O values of water samples were determined using a L1102-i water analyzer (Piccaro, Santa Clara, CA, USA). These samples were introduced into the vaporization chamber with the injection port using an attached PAL autosampler (CTC Analytics AG, Zwingen, Switzerland). Approximately 1 μL of water sample was injected into a heated vaporizer (140 °C) and then transferred to the cavity of the spectroscopic analyzer where the isotopologue concentrations were determined by cavity ring-down spectroscopy. Three international reference materials (SLAP, GISP and VSMOW) and an additional in-house reference material (calibrated against the VSMOW-SLAP scale) were included in each batch to correct the raw values via a three-point regression line (SLAP, GISP and VSMOW). The precision of the measurements was better than 1.4 ‰ for the *δ*^2^H values and 0.3 ‰ for the *δ*^18^O values.

## Results

### Direct measurement of tail hair growth rate in different equid species

The results from captive equids indicated inter-specific differences in average tail hair growth, with the tail hairs of domestic donkeys growing more than twice as fast as those of the two horse species (Table 2). Within species, tail hair growth showed considerable variation among individual Przewalski's horses and domestic donkeys, and was at the lower end of values previously reported for domestic horses and our Lipizzaner horse (Tables1 and 2). Although individual tail growth rates showed some variation between measurements, there was little indication that tail growth rates changed in any systematic manner in an individual or among individuals of the same species.

**Table 2:** Tail hair growth rate measurements (in mm/day) obtained from three different captive equid species in 2013–2014

Species	Domestic horse	Przewalski's horse	Domestic donkey
Mean all	0.57	0.57	1.18
SD	/	0.15	0.10
Range means all	/	0.50–0.65	1.07–1.25
N individuals	1	5	3
N measurements per individual	4	4–9	6–7

### Tail hair stable isotope patterns

#### Individual differences in tail hair growth in two individuals subject to identical, severe environmental change

**Figure 1:**
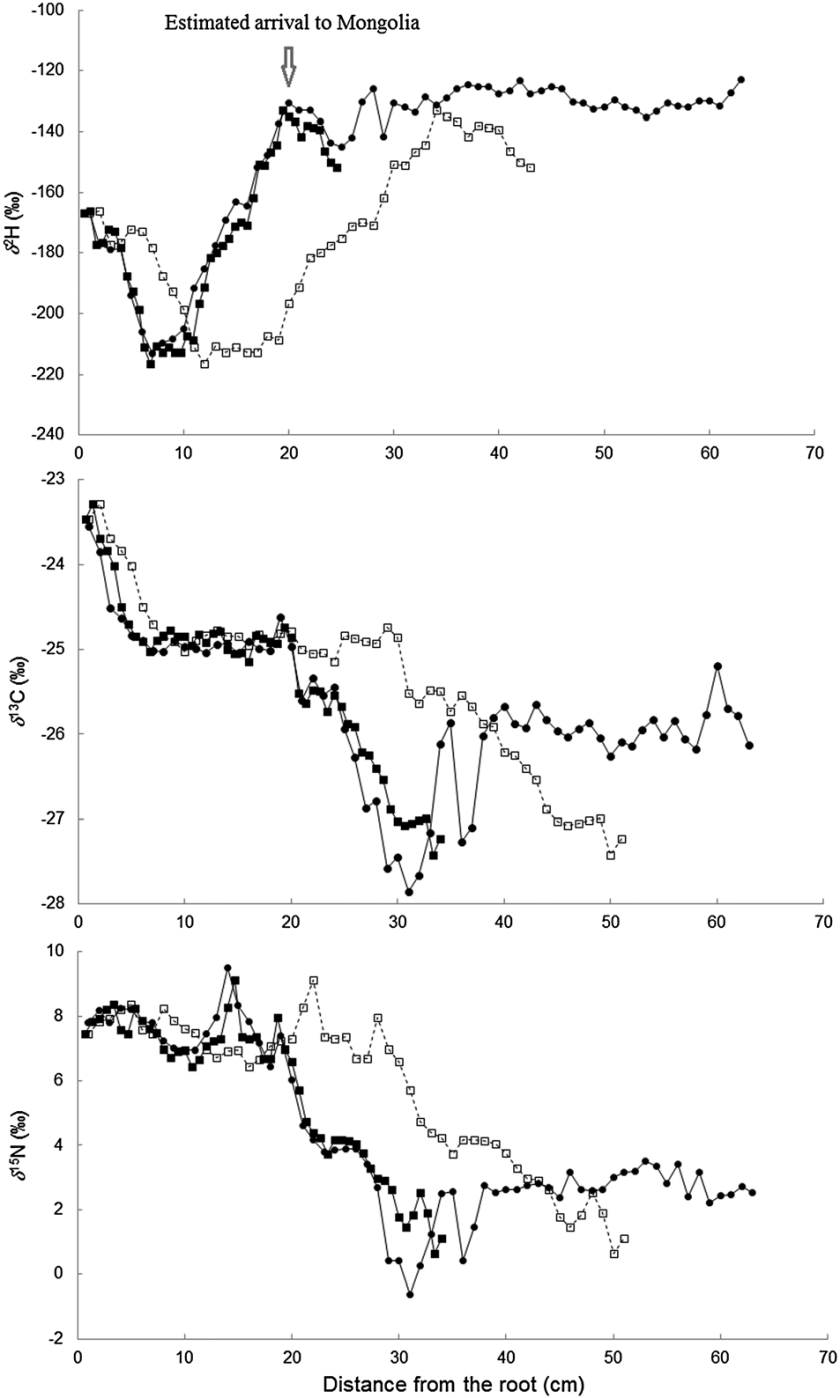
*δ*^2^H, *δ*^13^C and *δ*^15^N tail hair pattern of two Przewalski's horses, 13–053 (filled circles and solid line) and 13–057 (open squares and dashed line), subject to identical housing and feeding regimes. After correction for a different growth rate the isotope patterns of 13–057 (filled squares and solid line) closely match those of 13–053 for all three isotopes.

The two captive Przewalski's horses which had been transferred from Europe to Mongolia showed very similar isotope trends. However, plotted based on the distance from the root the isotopic signal frequencies did not correspond, but rather seemed compressed (or stretched depending on which animal was used as reference) which strongly suggested different tail hair growth rates in the two individuals (Fig.1). If not corrected for, the temporal shift becomes more and more pronounced the further one moves back in time from the sampling date (i.e. the root).

We corrected for different growth rates of the two animals using a visual (spreadsheet) matching approach, i.e. we visually aligned the isotope signatures on a spreadsheet by reducing the growth rate to compact chronology of sample 13–057. As reported by Wittemyer *et al*.,^12^ this approach gives comparable results to those derived from quantitative techniques. After reducing the growth rate of individual 13–057 to visually match individual 13–053, the isotope signatures of the two horses showed a high overall fit for *δ*^2^H, *δ*^13^C and *δ*^15^N values (Fig.1). The maximal *δ*-difference between measured minima and maxima of the two hairs was 0.8 ‰ for *δ*^13^C, 2.1 ‰ for *δ*^15^N and 9.5 ‰ for *δ*^2^H. Close correspondence of isotope signatures was indicated by high correlation coefficients and slopes of linear regression (r^2^ = 0.96, slope = 0.98 for *δ*^2^H; r^2^ = 0.87, slope = 0.86 for *δ*^13^C, and r^2^ = 0.92, slope = 0.90 for *δ*^15^N).^10^ This remarkable similarity indicates that both hairs were in the same growing phase at the time of sampling (anagen, i.e. active growing phase).^10^ We independently aligned H and CN isotope signatures as different hairs had been used for each analysis, but used the identical alignment (growth rate correction) for C and N because *δ*^13^C and *δ*^15^N values were analyzed simultaneously from the same hair and their isotope turnover rates (the pools and their relative half-lives for horse tail hair) should be identical (Thure E. Cerling, University of Utah, personal communication, 2014). With this example we want to point out the need to correct for differences in tail hair growth even in individuals subject to identical environmental change.

### Determining the correct time line for free-ranging equids

**Figure 2:**
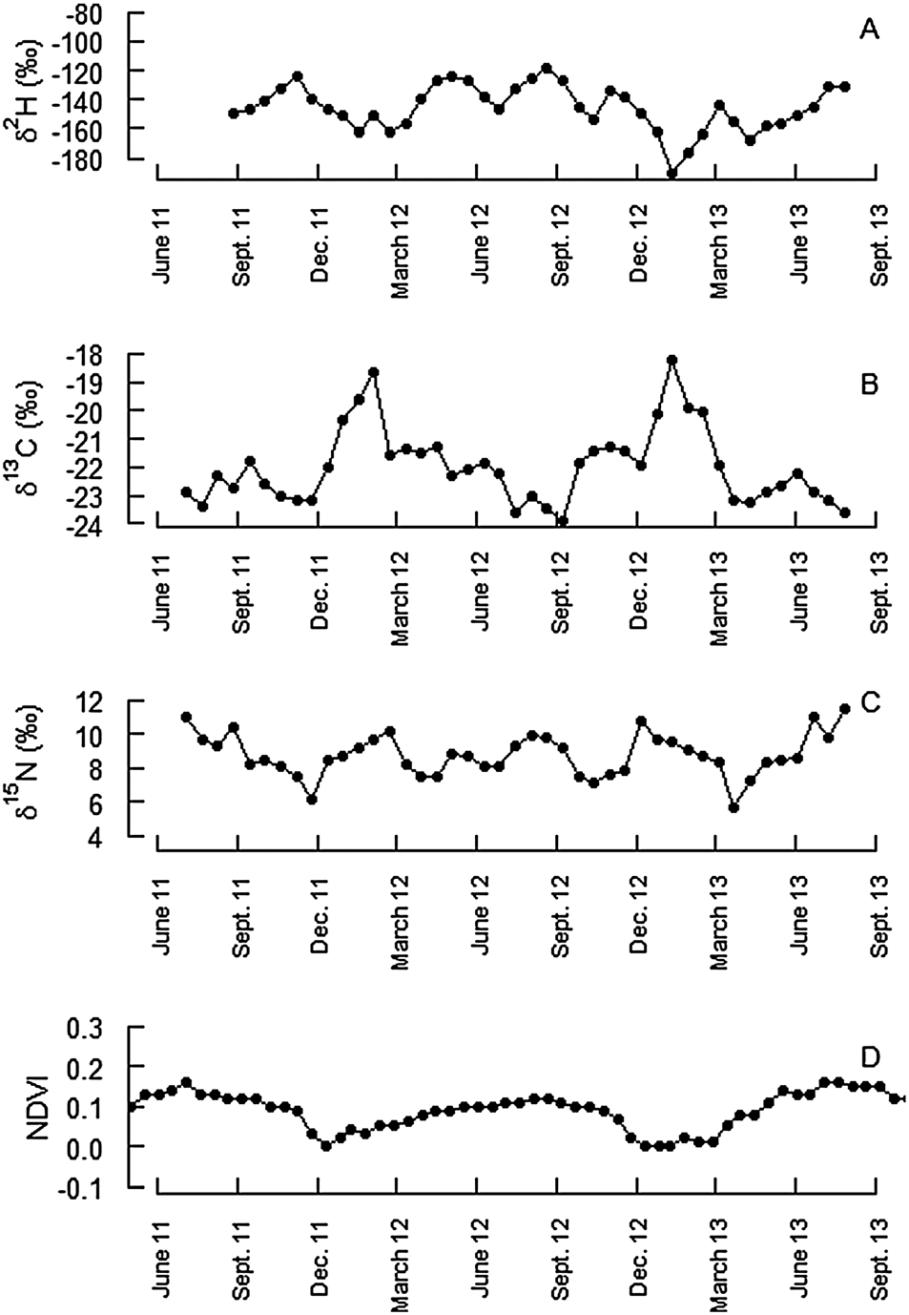
An example of *δ*^2^H (A), *δ*^13^C (B) and *δ*^15^N (C) primary tail hair patterns of an Asiatic wild ass assuming an annual interval between *δ*^13^C maxima and *δ*^2^H minima. The resulting time line of the newly aligned hair (assuming a constant growth rate) is consistent with winter lows and summer highs of the independent, temporally explicit NDVI values (D). (Sampling date of the Asiatic wild ass tail hair was 26.7.2013.)

The tail hair signatures of C and H isotopes showed distinct highs and lows in Asiatic wild asses with *δ*^13^C maxima corresponding to *δ*^2^H minima, and vice versa (Fig.2). The precipitation from the Great Gobi B SPA showed its lowest *δ*^2^H values during winter, i.e. between October and March, and higher *δ*^2^H values during summer (overall range of *δ*^2^H values >175 ‰; 2012–2013; Table 3). Previous observations suggested that Asiatic wild asses in the Gobi switch from visiting water points in the summer to eating snow in winter^29^ when the main water sources are frozen. Consequently, low *δ*^2^H values in the hair should be associated with winter and higher *δ*^2^H values with summer months and should present a yearly pattern (lows should be separated by 12 months). As the *δ*^13^C pattern in Asiatic wild asses follows an opposing trend, the high *δ*^13^C peaks should also be separated by 12 months, but should fall in the winter. Because the *δ*^13^C highs were more distinct than the *δ*^2^H lows, they provide an additional alignment option which was, however, only available for Asiatic wild asses, as Przewalski's horses and domestic horses failed to show a distinct and regular *δ*^13^C pattern (unpublished data).

**Table 3:** *δ*^2^H and *δ*^18^O values of precipitation, collected in the study area at the Takhin Tal research station between June 2012 and August 2013. Values are not weighted by the amount of precipitation

Sample no.	Date of sampling	Precipitation type	*δ*^18^O (‰)	δ^2^H (‰)
12-0666	15.06.2012	Rain	−7.5	−77.9
12-0667	16.06.2012	Rain	−10.8	−121.4
12-0668	22.06.2012	Rain	−9.9	−101.3
12-0669	23.06.2012	Rain	−13.7	−131.9
12-0670	25.06.2012	Rain	−11.3	−102.1
12-0840	08.07.2012	Rain	−11.8	−121.1
12-0841	22.07.2012	Rain	−5.8	−67.5
13-001	17.10.2012	Snow	−26.9	−226.8
13-002	21.10.2012	Snow	−26.1	−218.1
13-004	14.11.2012	Snow	−28.2	−235.9
13-005	04.12.2012	Snow	−30.0	−243.1
13-006	13.12.2012	Snow	−27.4	−231.2
13-007	09.01.2013	Snow	−27.0	−228.5
13-008	06.02.2013	Snow	−26.6	−222.2
13-009	07.02.2013	Snow	−26.5	−219.3
13-010	14.02.2013	Snow	−26.7	−224.2
13-011	25.02.2013	Snow	−27.0	−224.3
13-012	26.02.2013	Snow	−26.3	−217.8
13-013	06.03.2013	Snow	−22.0	−180.5
13-014	23.03.2013	Snow	−21.9	−180.4
13-015	21.04.2013	Rain	−13.3	−114.1
13-016	22.04.2013	Rain	−13.3	−114.1
13-155	19.06.2013	Rain	−11.3	−111.3
13-156	20.06.2013	Rain	−15.2	−125.7
13-158	30.07.2013	Rain	−5.8	−71.6
13-159	27.08.2013	Rain	−8.8	−76.4

To match the environmental seasonality, the tail hair growth rate was individually adjusted (by varying the assumed tail hair growth rate) so that the *δ*^2^H minima (and *δ*^13^C maxima) are spread apart by 12 months, and used the sampling date as explicit end data to create the corresponding time line (" 3-point adjustment" ). Subsequently, the time line was aligned with the corresponding temporally explicit NDVI values (Fig.2), and it was found that the *δ*^2^H minima (and *δ*^13^C maxima) coincided with the lowest NDVI values, indicative of snow or bare land, thus independently confirming our assumption that these *δ*^2^H minima and *δ*^13^C maxima represent winter.

**Figure 3:**
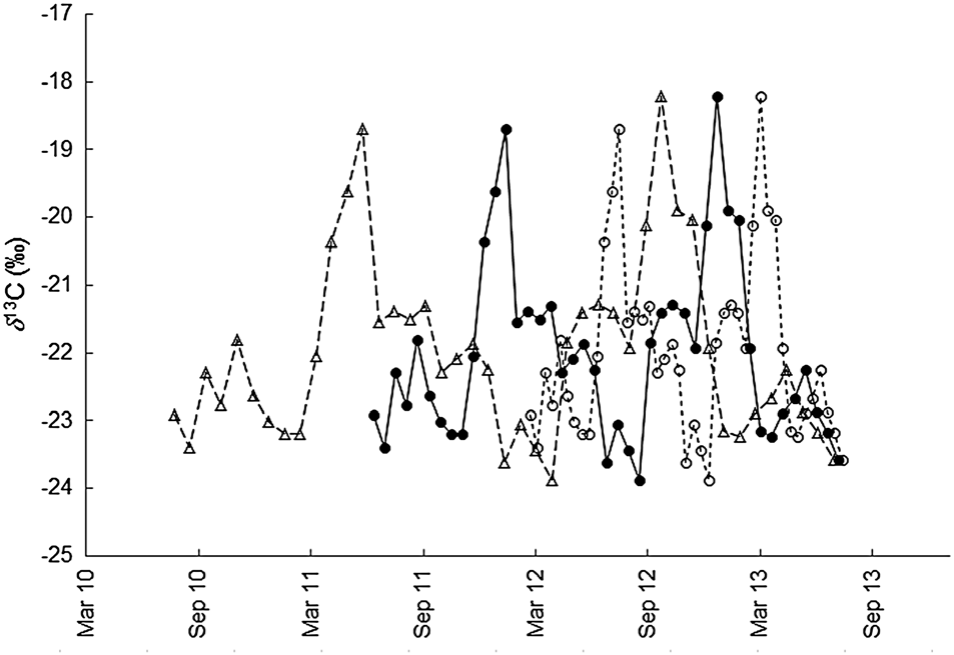
Temporal assignment of the Asiatic wild ass tail hair increments based on different tail hair growth rates: (a) assuming a 1-year interval between *δ*^13^C highs and *δ*^2^H lows corresponding to a growth rate of 0.52 mm/day (Table 4) (filled circles, solid line); (b) using the average growth rates of 0.79 mm/day obtained from domestic ponies in Wittmer^19^ (open circles, dotted line); and (c) using our average growth rate of 1.18 mm/day obtained from domestic donkeys (Table 2; open triangles, dashed line).

Using the known average tail hair growth rates of other equids for the temporal assignment, on the other hand, does not seem reasonable as it would assign the two highest *δ*^13^C values to August 2012 and March 2013 (7-month interval) when using the literature value for domestic ponies^29^ or to June 2011 and October 2012 (16-month interval) when using our own values for domestic donkeys (Table 2, Fig.3).

**Table 4:** Compilation of results of the tail hair growth rates (mm/day), indirectly calculated from the *δ*^13^C and *δ*^2^H isotope patterns

Species	Domestic horse	Przewalski's horse	Asiatic wild ass
Mean all	0.79	0.57	0.52
SD	0.11	0.06	0.06
Range	0.70–1.00	0.46–0.65	0.45–0.59
N of individuals	6	6	7

To address the intra- and inter-specific variation in tail hair growth we eventually adjusted the tail hair growth rate of each individual animal separately, following the procedure described above. For Asiatic wild asses, which showed a strong annual pattern for both *δ*^2^H and *δ*^13^C values, each individual's hair was aligned based on whether it was used for CN or H analysis. For Przewalski's and domestic horses which showed little seasonality in *δ*^13^C values, the tail hair growth rates were assigned based on the seasonal pattern of the *δ*^2^H results only.

The indirectly obtained growth rates (Table 4) for domestic and Przewalski's horses were all well within the range of our own direct measurements or published results, as was the variability among individuals. The values and variability in Asiatic wild asses differed greatly from those of domestic donkeys which have anatomically similar tails, but were very similar to those of Przewalski's horses (Tables 1 and 2).

## Discussion

Previous measurements of domestic horses,^13^,^30^–^32^ our own tail hair measurements of two additional equid species, and the comparative isotope analysis of two captive Przewalski's horses subject to identical and rather severe environmental changes confirmed that tail hair growth shows considerable inter- and intra-specific variation. Consequently, assuming equal growth rates for closely related species or even using the same known average tail hair growth rate for multiple individuals of the same species will probably result in incorrectly assigned time lines for single individuals or blurred pattern when pooled, thus potentially obscuring or dampening the peaks of existing seasonal patterns.

In regions which are subject to strong seasonal patterns we therefore suggest identifying key isotopes which show strong seasonal variation of known periodicity in the environment and which can be expected to be reflected in the animal tissue as a consequence of the species ecology. The known interval between maxima and minima of environmental isotope values can then be used to correctly temporally align the segmental stable isotope signature for each individual animal by using a growth rate which spreads maxima and minima to match the environmental interval. If the seasonality of one isotope pattern correlates with other isotopes, which show even more distinct maxima and minima (in our case C), those can be additionally used to refine the alignment.

The Mongolian Gobi with its extremely seasonal environment is ideal for this approach, particularly because all equids are water dependent^33^ and wild asses, Przewalski's horses and free-ranging domestic horses are known to switch from drinking liquid water in summer to eating snow in winter.^29^,^34^ Isotope measurements of precipitation showed the lowest *δ*^2^H values in winter and the switch in water sources was indeed reflected by a strong seasonal pattern of *δ*^2^H values in equid tails. Asiatic wild asses also showed an even more distinct seasonal pattern for C isotopes suggesting an annual dietary change with high *δ*^13^C values corresponding to low *δ*^2^H values. However, no such pattern was observed in Przewalski's and domestic horses nor had it been expected *a priori* and the same was true for the *δ*^15^N values in all three species.

Care has to be taken not to fall into the pit of circular reasoning by aligning hair to just any pattern without further baseline measurements of isotopic values (e.g. precipitation, soil, vegetation),^9^,^35^ and an understanding of the dynamics of the environment (e.g. via field surveys or remote sensing products like NDVI) and the species ecology. In addition, indirect tail hair growth rate obtained from aligned hair should not vary dramatically among individuals and/or be within the range of values reported in the literature.

We assumed a constant tail hair growth which is supported by a study of domestic horses that did not detect a change in tail hair growths throughout the year even in individuals exposed to a change from low- to high-protein feed.^31^ Whether this pattern also holds true for other species needs further investigation. At least in African elephants, tail hair growth seems to vary only slightly, even during periods of extreme metabolic stress (reproductive state).^12^ We did, however, observe variation in regrowth among measurements of the same individuals, but they seemed to occur at random and thus can be expected to level out if the time periods covered by the hair increments are not too short. In the case of our equids, 10-mm increments represent a period of 17–19 days in Przewalski's horses and Asiatic wild asses and about 13 days in domestic horses and our ambition was to draw inferences on seasonal effects manifesting themselves over the course of months. Hence, we believe that our approach presents a simple and robust protocol to correct for intra- and inter-specific variation in tail hair growth in order to align the isotope signatures of segmentally cut tail hair to a common time line, at least in highly seasonal environments. This protocol is also suitable for regions with a distinct dry and wet season (e.g. Namib Desert or the Serengeti Plains in Africa) or for animals that show large-scale migrations of known timing between different ecoregions (e.g. from predominantly C_3_ to C_4_ or to mixed C_3_/C_4_ biomes).
